# Pleomorphic giant cell carcinoma of the prostate: A case report and mini‑review of the literature

**DOI:** 10.3892/mi.2023.120

**Published:** 2023-11-10

**Authors:** Saman Salih Fakhralddin, Rawa M. Ali, Ari M. Abdullah, Farman Mohammed Faraj, Dlsoz M. Hussein, Shvan H. Mohammed, Berun A. Abdalla, Fahmi H. Kakamad, Hawbash M. Rahim

**Affiliations:** 1 College of Medicine, University of Sulaimani, Sulaimani, Kurdistan 46001, Iraq; 2 Department of Scientific Affairs, Smart Health Tower, University of Human Development, Sulaimani, Kurdistan 46001, Iraq; 3 Sulaimaniyah Surgical Teaching Hospital, University of Human Development, Sulaimani, Kurdistan 46001, Iraq; 4 Kscien Organization, University of Human Development, Sulaimani, Kurdistan 46001, Iraq; 5 Department of Medical Laboratory Science, College of Health Sciences, University of Human Development, Sulaimani, Kurdistan 46001, Iraq

**Keywords:** prostate cancer, pleomorphic giant cell carcinoma, prostate-specific antigen, Gleason grading, immunohistochemistry

## Abstract

Pleomorphic giant cell carcinoma (PGCC) is an exceptionally uncommon form of prostate adenocarcinoma. It consists of unusually large and irregular cells with varied nuclei. The present study describes a rare case of prostatic PGCC. A 65-year-old male patient presented to the urology clinic with severe dysuria, nocturia, and frequent, urgent, and difficult urination for a period of 3 months. Pelvic magnetic resonance imaging revealed a large pelvic mass. A prostate biopsy was performed, and immunohistochemical analysis revealed positivity for the pan-epithelial markers, AE1/AE3, alpha-methyl acyl-CoA racemase, and focally for sphingolipid activator protein-2. While waiting for his pathology report, the patient's condition deteriorated, and he was diagnosed with intestinal obstruction. The patient underwent laparotomy and end colostomy. Later, he developed severe sepsis and wound dehiscence. After 2 weeks, the patient succumbed due to multiorgan failure. Prostatic PGCC cases are frequently associated with previous chemo-, hormone, or radiation therapy. Prior to the diagnosis of PGCC, it is critical to rule out urothelial carcinoma. Early recognition of this rare condition can lead to more effective therapy. Prostatic PGCC is extremely rare. Immunohistochemistry for prostatic markers, such as prostate-specific membrane antigen, prostate-specific antigen, NK3 homeobox 1 and androgen receptor, can be used to confirm its origin.

## Introduction

Prostatic cancer is one of the most highly prevalent malignancies affecting males globally (1). The spectrum of prostatic neoplasms includes a variety of rare histological variants. Among these, prostatic pleomorphic giant cell carcinoma (PGCC) is an extremely rare and poorly understood subtype, recently listed in the World Health Organization (WHO) Classification of Tumors of the Urinary System as a variant of acinar adenocarcinoma (2,3). Based on current data, PGCC is an aggressive form of acinar adenocarcinoma associated with a poor prognosis, despite treatment (4). PGCC typically demonstrates a small portion of more differentiated, yet high-grade, typical prostatic acinar adenocarcinoma (5). It is characterized by giant, bizarre cells with pleomorphic nuclei upon a conventional histological examination (2,3,6).

Some PGCC cases may also contain components of other tumor types, such as squamous cell carcinoma, neuroendocrine carcinoma and prostatic ductal adenocarcinoma (2). When compared to conventional prostate carcinoma, PGCC frequently exhibits high Gleason grade characteristics and currently falls into the International Society of Urological Pathology (ISUP) grade group 5 (3,6,7), based on the recent 5th edition WHO classification of male genital and urinary tumors (2). Moreover, previous chemotherapy, hormonal therapy and radiation therapy are commonly related to occurrences of PGCC, particularly androgen deprivation therapy (3,8). Given its rarity, prostatic PGCC often presents significant diagnostic and treatment challenges, exacerbated by its aggressive nature and poor prognosis. The scarcity of prostatic PGCC case reports in the medical literature underscores the need for more comprehensive data on this rare pathologic entity. The present study reports a case of a 65-year-old male patient with prostatic PGCC and also provides a brief review of the literature with the aim of shattering further light on this rare entity.

## Case report

### Patient information

A 65-year-old male patient visited Smart Health Tower (Sulaimani, Iraq) and complained of severe dysuria, nocturia, and frequent, urgent, and difficult urination for a period of 3 months. He had previously visited numerous urologists, and they diagnosed his condition as benign prostatic hypertrophy with prostatitis. He developed acute urinary retention while on medical treatment for prostatitis. An 18 French Foley catheter was inserted for him for 1 week; however, following its removal, the patient was unable to urinate again. Consequently, the Foley catheter was inserted again and kept for 2 months, being changed once every 2 weeks. Despite receiving multiple different types of antibiotics and alpha-blockers, his condition remained the same. He then complained of a generalized body ache, weakness, anorexia, weight loss, constipation, back pain and insomnia with severe lower urinary tract symptoms (LUTS).

### Clinical examination

Upon an examination, the patient was found to be pale, have a low body weight, and have a soft abdomen with tenderness over the lower abdomen. His urine bag contained turbid urine. A digital rectal examination revealed a significantly enlarged and painful prostate bulging into the rectum, with a smooth surface and no nodularity. The upper portion could not be reached due to its large size.

### Diagnostic assessment

**Figure 1:**
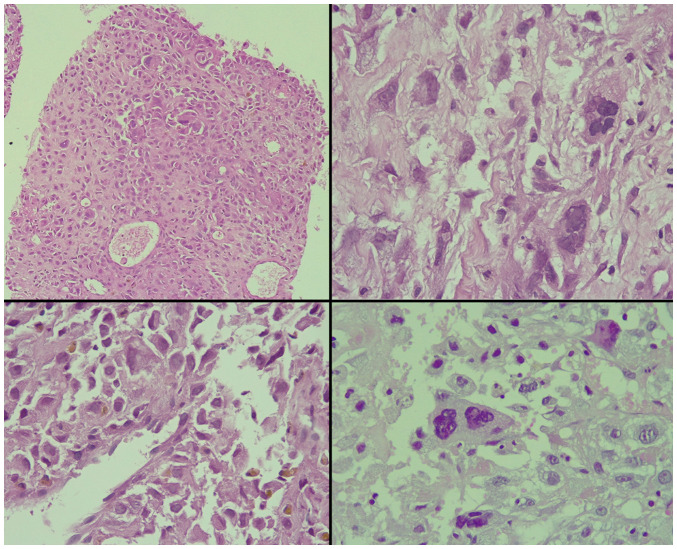
Microscopic images illustrating sheets of large cells with an abundant eosinophilic cytoplasm and widely pleomorphic, bizarre and hyperchromatic nuclei with irregular nuclear outlines and frequent multinucleation (hematoxylin and eosin staining). Magnification: Upper left panel, x100; other panels, x400.

**Figure 2:**
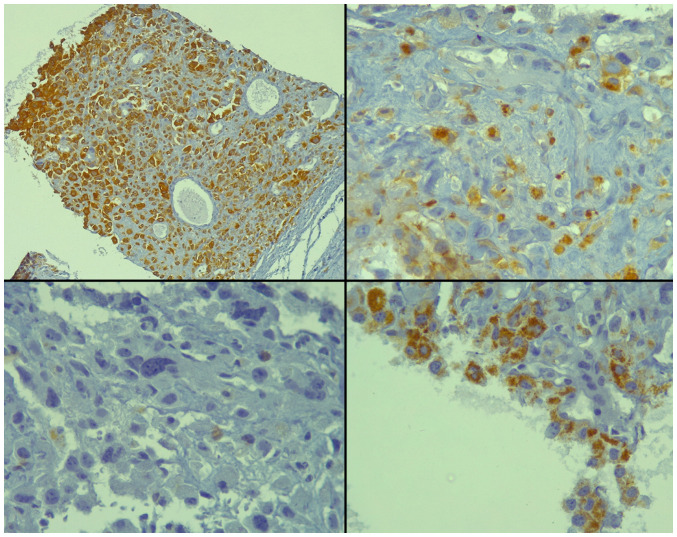
Immunohistochemical analysis of the core biopsy illustrating strong and cytoplasmic staining for the pan-keratin marker, AE1/AE3 (upper left panel), negativity for PSA (lower left panel), patchy and strong cytoplasmic staining for PSAP (upper right panel), and patchy and strong granular cytoplasmic staining for AMACR (lower right panel). Immunohistochemistry was performed using diamainobenzidine chromogen. Magnification: Upper left panel, x100; other panels, x400.

Laboratory investigations, including a complete blood count, renal function tests and prostate-specific antigen (PSA), were performed and all yielded normal results. The patient was sent for pelvic magnetic resonance imaging (MRI) for more detailed information about the prostate and pelvic organs. The report of the pelvic MRI revealed a large prostatic mass measuring 9x9x11 cm with a well-defined outline and multiple areas of cystic degeneration. Some of the cysts exhibited fluid-fluid levels (indicating variably-aged internal hemorrhage). The mass originated from the prostate with a marked pressure effect on the rest of the prostate, urinary bladder and rectum. No definite invasion to the surrounding organs and no associated pelvic lymphadenopathy were observed. There was a focal bone lesion involving the left pubic bone, suggestive of bone metastasis. A well-defined enhancing mass was located in the left gluteal region under the gluteus maximus muscle measuring 16x11 mm. The urinary bladder wall was thin and there was no ascites. The case was presented to a multidisciplinary team, which included urologists, general surgeons, radiologists, pathologists and uro-oncologists; the decision was to perform a prostate biopsy for tissue diagnosis. Following preparation, the patient underwent a transrectal, 12-core prostate biopsy. Upon a histopathological examination, it was found that 80% of the tissue involved by the tumor had a Gleason score of 10 (5+5) for conventional prostate carcinoma, composed of dyscohesive, large, and pleomorphic cells with abundant eosinophilic cytoplasm and bizarre, hyperchromatic nuclei with irregular nuclear outlines, intranuclear inclusions, large macronucleoli and multinucleation (Fig. 1). Perineural invasion was observed; however, intraductal carcinoma, extraprostatic extension and lymphovascular invasion were not observed. An immunohistochemical analysis revealed positivity of the tumor cells for the pan-epithelial markers AE1/AE3 and alpha-methylacyl-CoA racemase (AMACR), and focal positivity for sphingolipid activator protein-2 (SAP) (Fig. 2). Of note, all staining protocols and immunohistochemistry were conducted in an external facility and not at the authors' institute. All the other markers studied were negative, including PSA, melanin A, desmin, CD34, CK7, CK20, caudal type homeobox 2 (CDX2), GATA binding protein 3 (GATA3) and thyroid transcription factor-1 (TTF-1) (data not shown). Hence, the diagnosis of PGCC of the prostate was made.

### Therapeutic intervention and follow-up

While waiting for the results of the histopathological examination of the prostate biopsy, the patient developed acute abdominal distension and pain with repeated vomiting, for which he was admitted to the *emergency room* and was diagnosed with intestinal obstruction. Following 2 days of conservative treatment (as at that time, the family refused any surgical intervention, the gastrointestinal tract surgeon was obliged to manage the patient by inserting a nasogastric tube, antibiotics and IV fluid), the gastrointestinal tract surgeon decided to do a laparotomy and end colostomy for him. After 4 days, his condition deteriorated, and he developed severe sepsis with wound dehiscence. He was admitted to the intensive care unit, and after 2 weeks of follow-up, the patient passed away from multiorgan failure.

## Discussion

PGCC, a relatively rare tumor variant, has been identified in several organs, including the hepatobiliary system, pancreas, thyroid, urinary bladder, endometrium and kidney (3,5,9). In the context of the prostate, the first PGCC case was documented by Mai *et al* in 1996(10). This variant of carcinoma is interesting due to its unique histological presentation and diagnostic challenges, particularly in differentiating it from other forms of cancer.

Standard prostate cancer generally exhibits cells that possess relatively uniform nuclei, even in high-grade cases (7). This lack of pleomorphism serves as a key characteristic that sets poorly differentiated prostate cancer apart from urothelial carcinoma and allows for differentiation from sarcomatoid carcinoma due to the absence of spindle cells (3,5). However, in uncommon cases, the histological features of pleomorphic giant cell adenocarcinoma of the prostate can overlap with those of urothelial carcinoma, which poses a diagnostic challenge, given that the treatments for these two diseases are markedly different.

Clinically, determining whether a sizable tumor at the bladder neck is of bladder or prostatic origin can be difficult on imaging and even during cystoscopy. Experienced urologists have submitted numerous cases of ‘bladder tumors’ that were initially misdiagnosed by pathologists as urothelial carcinomas. Upon further review and the implementation of immunohistochemistry, these tumors were identified as high-grade prostatic adenocarcinomas (7). Previous reports of prostatic PGCC cases (7,11) have detailed similarities to conventional prostate cancer, including an aggressive clinical course, an increased incidence in older patients, an association with a high Gleason grade for standard prostate carcinoma components, and typically focal, negative, or weak staining for standard prostatic immunohistochemical markers (3).

Pleomorphic giant cell adenocarcinoma has very particular histological features. The tumor cells display marked pleomorphism and varying levels of cohesiveness. Even in the presence of extreme atypia, more conventional features can still be observed. If PGCC is associated with a more traditional acinar adenocarcinoma, the latter component generally exhibits a high Gleason grade, thereby categorizing this unique form of prostate cancer under ISUP grade group 5, even in the absence of more classical features (2,6,12). In terms of morphology, PGCCs consist of unusually large cells that typically comprise a small fraction of the overall tumor. These cells are distinctively epithelial and form a cohesive structure, which aids in distinguishing them from the diverse, pleomorphic individual cells seen in sarcomatoid prostate adenocarcinoma (7). Atypical mitoses are frequently observed, and necrosis has been documented in the literature, with the case reported by Larnaudie *et al* (13) illustrating hemorrhagic and necrotic features. Another common observation is the clearing of the cytoplasm, while spindle cell aspects have been described, albeit being rare, as reported by Larnaudie *et al* (13). PGCC has been shown to be related to other histological variants of prostatic carcinoma, including squamous cell carcinoma, intraductal carcinoma and neuroendocrine carcinoma (13). In the six cases documented by Parwani *et al* (5), each one exhibited an additional component of either small cell, squamous cell, or prostatic ductal carcinoma. The tumor in the patient depicted herein displayed clusters of loosely cohesive cells characterized by abundant eosinophilic cytoplasm and large, hyperchromatic nuclei with intranuclear inclusions, sizable macronucleoli and multinucleation.

Diagnostic difficulties may arise when only the pleomorphic component is present. The ability to recognize the prostatic origin of the tumor is crucial, particularly in cases of isolated metastasis, as the treatment modalities diverge substantially, with one leaning towards hormonotherapy and chemotherapy (13). To confirm the origin of PGCC, the use of prostatic marker immunohistochemistry, such as prostate-specific membrane antigen (PSMA), PSA, NK3 homeobox 1 (NKX3.1) and androgen receptor, is recommended, as previously suggested by Alharbi *et al* (7) and corroborated by Bilé-Silva *et al* (4).

El-Zaatari *et al* (3) emphasized the importance of a comprehensive panel of immunohistochemical markers for correct PGCC diagnosis. Their review of 51 PGCC cases revealed that numerous cases exhibited weak or no staining for any marker of prostatic differentiation. Intriguingly, despite being commonly performed, PSA staining was positive in only 2 out of 22 cases, whereas 10 cases exhibited weak and focal staining and the others were entirely negative (3). The study by Bilé-Silva *et al* (4) also revealed a lower PSA positivity, ranging from 5 to 20%. However, this low expression should not be construed as a negative PSA expression, as it may result from various therapies (4). NKX3.1 emerged as a superior prostate-specific marker for distinguishing urothelial carcinoma and poorly differentiated prostatic adenocarcinoma, exhibiting a high sensitivity and specificity for the latter (14). Yet, Alharbi *et al*.'s study showed that its staining was not consistent, with some cases showing only focal positivity or even negativity in the pleomorphic component (7). Another promising marker is homeobox B13 (HOXB13), exhibiting a high sensitivity and specificity for prostatic tissue. A 2017 study suggested its utility in confirming the prostatic origin of metastatic lesions (13). However, the study by Alharbi *et al* (7) revealed that HOXB13 staining varied significantly among PGCC cases, with some cases even showing complete negativity. The cases reported in the study by Parwani *et al* (5) were all positive for cytokeratin AE1/AE3. Lastly, the majority of the PGCC cases in the study by Alharbi *et al* (7) exhibited negativity for GATA3, p63, and thrombomodulin, which is useful for ruling out urothelial carcinoma. All these findings emphasize the necessity for a comprehensive panel of markers to ensure an accurate diagnosis of PGCC. The case in the present study was positive for PSAP, AMACR, and AE1/AE3, while being negative for PSA, CK7, CK20, GATA3, melan-A, TTF-1, desmin, CD34 and CDX2.

Prior radiation, hormonal therapy, and chemotherapy appear to be frequently associated with the PGCC phenotype (4,8,15). There is substantial evidence to indicate that PGCC develops as a result of the dedifferentiation of high-grade, traditional prostate carcinoma. The conventional prostatic adenocarcinoma component has coexisted with all known PGCC cases. Notably, 11 out of 12 cases with earlier traditional prostatic cancer, including the cases reported in the study by El-Zaatari *et al* (3,5,6), had undergone radiation, chemo-, or hormonal therapy prior to presenting with PGCC. This suggests that prior treatment modalities may contribute to PGCC development (3,5,7). This is supported by observations of the frequent loss/weak staining of prostatic differentiation markers in these tumors, pointing towards a loss of prostatic differentiation (3). Further confirming this theory, the study by Bilé-Silva *et al* (4) demonstrated that all the patients with PGCC had previously been treated with hormonal therapy.

The most recently published study by Bilé-Silva *et al* (4), which included the largest series of patients with extensive PGCC, provides a comparative survival analysis between cases with PGCC and cases with conventional high-grade prostate adenocarcinoma. The findings indicate a significant difference in cancer-specific survival, suggesting the aggressive nature of PGCC (4). This is also supported by the findings of the cases reported by Alharbi *et al* (7).

Further research is required in order to confirm these findings. Improving the understanding of this variant of prostate cancer could allow for an earlier and more accurate diagnosis, leading to more effective therapeutic approaches that could potentially enhance patient outcomes.

In conclusion, PGCC of the prostate is an aggressive variant with a dismal prognosis. It frequently occurs in patients who have received prior prostatic cancer-directed therapy. Prostatic marker immunohistochemistry, such as PSMA, PSA, NKX3.1 and androgen receptor can be used to confirm whether the PGCC is of prostatic origin. The early recognition of this entity may contribute to more effective therapy, as physicians could opt for more aggressive treatments.

## Data Availability

The datasets used and/or analyzed during the current study are available from the corresponding author on reasonable request.
